# Follow-up of colorectal cancer patients: quality of life and attitudes towards follow-up.

**DOI:** 10.1038/bjc.1997.161

**Published:** 1997

**Authors:** A. M. Stiggelbout, J. C. de Haes, R. Vree, C. J. van de Velde, C. M. Bruijninckx, K. van Groningen, J. Kievit

**Affiliations:** Medical Decision Making Unit, Leiden University Hospital, The Netherlands.

## Abstract

The aims of our study were to assess the effect of follow-up on the quality of life of colorectal cancer patients and to assess the attitudes of patients towards follow-up as a function of patient characteristics. Patients who had been treated with curative intent were selected from four types of hospitals. Eighty-two patients were interviewed using a structured questionnaire, whereas 130 patients received the questionnaire by mail. To assess the effect of follow-up on the quality of life, the interviewed patients were randomly allocated to three groups and interviewed at different times in relation to the follow-up visit. Analysis did not show an effect of the follow-up visit on quality of life. Patients reported a positive attitude towards follow-up: it reassured them, they judged the communication with the physician to be positive, and they experienced only slight nervous anticipation and few other disadvantages. Patients reported a strong preference for follow-up, and a large majority would prefer follow-up even if it would not lead to earlier detection of a recurrence. Apart from living situation, no patient characteristics were clearly associated with the attitude towards follow-up. Implications for clinical practice are discussed.


British Journal of Cancer (1997) 75(6), 914–920
© 1997 Cancer Research Campaign

### Follow-up of colorectal cancer patients: quality of life and attitudes towards follow-up

**AM Stiggelbout[1], JCJM de Haes,[1,2] R Vree[3], CJH van de Velde[4], CMA Bruijninckx[5], K van Groningen[6] and J Kievit[1,4]**

[1]Medical Decision Making Unit, Leiden University Hospital, PO Box 9600, 2300 RC, Leiden; [2]Department of Medical Psychology, Academic Medical Center, Meibergdreef 15, 1105 AZ, Amsterdam; [3]Department of Surgery of the Diaconessen Hospital, PO Box 9650, 2300 RD, Leiden [4]Department of Surgery, Leiden University Hospital, PO Box 9600, 2300 RC, Leiden; [5]Department of Surgery and [6]Department of Pathology of the Leyenburg Hospital, PO Box 40551, 2504 LN, The Hague, The Netherlands

**Summary** The aims of our study were to assess the effect of follow-up on the quality of life of colorectal cancer patients and to assess the attitudes of patients towards follow-up as a function of patient characteristics. Patients who had been treated with curative intent were selected from four types of hospitals. Eighty-two patients were interviewed using a structured questionnaire, whereas 130 patients received the questionnaire by mail. To assess the effect of follow-up on the quality of life, the interviewed patients were randomly allocated to three groups and interviewed at different times in relation to the follow-up visit. Analysis did not show an effect of the follow-up visit on quality of life. Patients reported a positive attitude towards follow-up: it reassured them, they judged the communication with the physician to be positive, and they experienced only slight nervous anticipation and few other disadvantages. Patients reported a strong preference for follow-up, and a large majority would prefer follow-up even if it would not lead to earlier detection of a recurrence. Apart from living situation, no patient characteristics were clearly associated with the attitude towards follow-up. Implications for clinical practice are discussed.

**Keywords:** colorectal cancer; routine follow-up; quality of life; patient preferences

Following curative surgery for colorectal cancer, most patients are submitted to some form of oncological follow-up. The main purpose of this follow-up is to detect recurrences and metachronous tumours in an early phase, when curative treatment may still be an option. Additional reasons for follow-up may be quality control for the surgeon and support of the patient (Bruinvels, 1995). The effectiveness and efficiency of oncological follow-up have become more and more a subject of debate (Deveney and Way, 1984; Sugarbaker et al, 1987; Isbister, 1988; Loprinzi, 1995; Virgo et al, 1995). Important effects to be considered in determining appropriate follow-up practices include longevity, quality of life and financial implications (Loprinzi, 1995). Effects on longevity are unclear. Although aggressive surveillance undoubtedly detects some cancers before symptoms develop, it is uncertain whether survival is measurably affected (Virgo et al, 1995). In a recent meta-analysis of studies comparing intensive follow-up with minimal or no follow-up, no statistically significant difference in survival was found (Bruinvels et al, 1994).

Little is known about the effects of routine follow-up on the quality of life of colorectal cancer patients and about the value that patients attach to follow-up. Breast cancer patients have been found to experience more psychological complaints and fear of recurrence just before the follow-up visit (Broyn and Froyen, 1982; Rutgers, 1986). On the other hand, follow-up may have a positive effect on feelings of security, and the visits may provide reassurance. In an earlier study from our institute, patients were found to experience reduced physical and psychological distress 2 weeks after a follow-up visit compared with immediately preceding the visit or 1 month beforehand (Kiebert et al, 1993). The majority of these patients had a diagnosis of breast cancer. In a recent large randomized trial in breast cancer, evaluating the effect of intensive compared with minimalist follow-up, no differences in health-related quality of life were seen between the two strategies (GIVIO investigators, 1994). Moreover, neither an increased anxiety nor stronger reassurance were seen in the intensive follow-up group. Both the study of Kiebert et al (1993) and the GIVIO study reported that patients had a strong preference for routine visits. Most studies on the effects of follow-up pertain to breast cancer (see also Broyn and Froyen, 1982; Rutgers, 1986). The value of oncological follow-up in colorectal cancer may differ from that in breast cancer. Whereas cure in case of metastasized breast cancer is impossible, surgery with curative intent or long-term control may still be possible in case of hepatic metastases from colorectal cancer. Given this potential advantage of colorectal cancer follow-up, it is of interest to assess the attitudes of colorectal cancer patients towards follow-up. Insight into factors that are associated with a positive or negative attitude, or that influence feelings of anxiety or reassurance, may help in determining the best follow-up schedule for colorectal cancer patients.

The first purpose of our study was to assess the effect of the follow-up visit on patients' quality of life. We hypothesized that patients interviewed shortly after the follow-up visit would have fewer psychological and physical complaints than patients interviewed immediately before follow-up or halfway between two visits (Kiebert et al, 1993). The second purpose was to assess the attitudes of colorectal cancer patients towards oncological follow-up and their strength of preference for follow-up and to see whether these are associated with patient characteristics (such as sociodemographics, medical history and quality of life).

*Received 13 June 1996*
*Revised 10 October 1996*
*Accepted 15 October 1996*

*Correspondence to:* AM Stiggelbout

### MATERIALS AND METHODS

### Patients

To obtain generalizability, patients were selected from four different hospitals: a university hospital (Leiden University Hospital), a small hospital in a large town (Diaconessenhuis, Leiden), a large inner-city hospital (Leyenburg, The Hague) and a large regional hospital (Medical Center, Alkmaar). All patients had been treated at or were in the follow-up protocol of one of the four hospitals, were free of disease, had had at least two follow-up visits (in order to be able to express an opinion on follow-up at the time of the study) and had been treated no more than 5 years before. In the two Leiden hospitals, patients were selected from the outpatient clinic appointment schedules. To assess whether our samples were selective, we cross-checked the appointment schedules with the hospital oncology databases to evaluate whether we had missed patients that were lost to follow-up. In the Leiden University Hospital only four patients were found that should have been in follow-up, but were not. We interviewed those patients to determine whether they had a different quality of life or attitude towards follow-up. As this was not the case, we excluded their data from this study as it pertains to an evaluation of follow-up. In the Diaconessenhuis, in which we interviewed only a sample of all cases, we randomly selected 25 cases from the oncology database that were not in the appointment system. For all but one case – for whom the patient file had been lost – there were legitimate reasons why the patient did not have an appointment (moved out of the region, too old, recurrent cancer, etc.). Thus, we feel confident that the patients interviewed reflect a population of colorectal cancer patients submitted to follow-up. In the other two hospitals, patients were selected either from the hospital oncology database (Medical Center, Alkmaar) or from the pathology database PALGA/Dutch Network & National Database for Pathology (Leyenburg Hospital, The Hague).

### Procedures

#### Elicitation mode

In the Leiden University Hospital and Diaconessenhuis, Leiden, interviews were held, in the context of a larger study assessing the effectiveness of follow-up in colorectal cancer (Stiggelbout et al, 1995). In the Leyenburgh Hospital, The Hague, and the Medical Center, Alkmaar, only data on patient attitudes and quality of life were obtained. These could be collected by means of questionnaires. To assess whether the results of the interviews might differ from those of the questionnaires, we mailed questionnaires to an additional sample of patients who were from the two hospitals at which we had conducted the interviews.

#### Design

To assess the effect of the follow-up visit per se on the quality of life of patients, we compared three patient groups who were interviewed at different times. For this purpose, we randomly divided the interviewed patients into three subgroups, i.e. those interviewed (a) 1 week before a scheduled follow-up visit, (b) 2 weeks after a follow-up visit, and (c) in the middle between two follow-up visits. This procedure was not possible for the patients that received mailed questionnaires. Data from these patients are therefore used only to assess the attitudes towards and strength of preference for follow-up.

#### Quality of life

Data were obtained using the Medical Outcomes Study short-form general health survey (MOS SF-20; Stewart et al, 1988) and the psychological and physical distress scales of the Rotterdam Symptom Checklist (RSCL; De Haes et al, 1990). The MOS SF-20 consists of 20 items covering six dimensions: health perceptions, physical functioning, mental health, social functioning, role functioning and pain. The RSCL was developed specifically for cancer patients. It contains a list of 30 items to assess the physical and psychological distress experienced by the patient in the preceding week. Finally, the patients rated their overall quality of life during the preceding week by means of a visual analogue scale (a 100 mm horizontal line, anchored at the extremes by 'best imaginable quality of life' and 'worst imaginable quality of life').

Within the format of the RSCL, three questions were posed assessing fear of recurrence (see Table 1). These items, also used in the former study at our hospital (Kiebert et al, 1993), formed a separate factor with satisfactory reliability (Cronbach's alpha = 0.75).

#### Attitudes towards follow-up

A 16-item questionnaire was developed based on previous research (Kiebert et al, 1993; see Appendix for details of questionnaire construction). This follow-up questionnaire consisted of four subscales: communication (with the physician), reassurance, nervous anticipation and specific perceived disadvantages of follow-up. The items and scales are given in Table 2. The following reliabilities were found: Cronbach's alpha for the disadvantages scale was 0.45, for the reassurance scale 0.66, for the anticipation scale 0.71 and for the communication scale 0.81.

#### Strength of preference for follow-up

In the interviews, an additional preference question was posed. (For examples of treatment trade-off or treatment preference methods, see Llewellyn-Thomas et al, 1989; Boyd et al, 1990.) The subject was asked to imagine a hypothetical choice between follow-up or no follow-up. It was stressed to the patients that the situation was

**Table 1** Fear of recurrence: items of the scale[a] and distribution of the scores [number (%)]

| | Not at all | Somewhat/to some extent | Rather | Very much |
|---|---|---|---|---|
| Do you feel insecure about your health? | 80 (38) | 96 (46) | 29 (14) | 4 (2) |
| Do you think the disease might still recur? | 46 (24) | 107 (55) | 29 (15) | 11 (6) |
| Do you feel completely cured? | 63 (31) | 78 (38) | 45 (22) | 17 (8) |

[a]Cronbach's alpha for the scale was 0.75.

© Cancer Research Campaign 1997 

**Table 2** Cancer patients' attitudes towards follow-up: the items of the questionnaire and the distribution of the scores [*n* (%)]

| | Factor[a] | Not at all | Somewhat/to some extent | Rather | Very much |
|---|---|---|---|---|---|
| Do the follow-up visits convey you a sense of security? | R | 11 (5) | 34 (16) | 80 (38) | 84 (40) |
| Are you nervous before a follow-up visit? | A | 107 (51) | 60 (29) | 35 (17) | 8 (4) |
| Are you reassured after the follow-up visit? | R | 8 (4) | 34 (16) | 82 (39) | 84 (40) |
| Do you sleep less well in the week before follow-up? | A | 164 (79) | 28 (13) | 15 (7) | 2 (1) |
| Would you prefer your family physician to perform the follow-up? | D | 153 (76) | 17 (8) | 15 (7) | 17 (8) |
| Can you ask about things at follow-up? | C | 17 (8) | 31 (15) | 85 (42) | 72 (35) |
| At follow-up, can you discuss with your doctor matters that are of concern to you or about which you worry? | C | 14 (7) | 29 (14) | 80 (40) | 78 (39) |
| Do you postpone plans till after the follow-up visit? | A | 148 (72) | 26 (13) | 22 (11) | 11 (5) |
| Do you think the investigations at follow-up burdensome? | D | 118 (57) | 56 (27) | 20 (10) | 15 (7) |
| Do the advantages of follow-up outweigh the disadvantages? | R | 18 (9) | 25 (12) | 67 (33) | 94 (46) |
| Would you worry more about your disease if there were no follow-up? | R | 22 (11) | 36 (17) | 59 (28) | 92 (44) |
| Do people in the hospital pay attention to what you say? | C | 5 (3) | 11 (5) | 104 (51) | 83 (41) |
| Do you normally dread the follow-up visits? | A | 122 (58) | 47 (23) | 31 (15) | 9 (4) |
| Does the follow-up remind you each time of your disease, while you'd rather think less often about it? | D | 97 (46) | 61 (29) | 37 (18) | 14 (7) |
| Would you rather have follow-up visits less frequently? | A | 149 (74) | 28 (14) | 14 (7) | 10 (5) |
| Do the physicians at follow-up in the hospital have enough time for you? | C | 11 (5) | 11 (5) | 120 (59) | 61 (30) |

[a]Factors: C, communication; A, nervous anticipation; R, reassurance; D, general disadvantages.

hypothetical, that the true figures were unknown and that the question was only aimed at obtaining a measure of their strength of preference for follow-up. In the 'follow-up' strategy, the chance of detecting a recurrence in an early stage (in which treatment was still possible) would be larger than in the 'no follow-up' strategy (initial values of 80% and 40% respectively). Subsequently, the chance of early detection in the 'no follow-up' strategy was increased stepwise to assess at what (if any) chance the respondent would switch to the 'no follow-up' strategy. (If a subject initially preferred the 'no follow-up' strategy, the chances of early detection in this strategy were decreased to assess at what chance he or she would switch to 'follow-up.') Thus an impression could be obtained of the strength of preference of the patient for follow-up.

### *Sociodemographic data and medical history*

Data were collected pertaining to sociodemographics (age, sex, education, living situation), date of diagnosis (or curative re-resection), frequency of follow-up and co-morbidity (yes/no).

### Data analysis

Scores on the items of the psychological and physical distress scale of the RSCL were coded from 0 to 3 and summed. Scores on the MOS-SF20 were summed as reported by Stewart et al, (1988) and rescaled from 0% to 100%. A higher score indicates better functioning for all dimensions but pain, for which a higher score indicates more pain. Scores on the follow-up questionnaire were summed (unweighted) and rescaled from 0% to 100%. For the communication and the reassurance scales, a higher score meant a more positive evaluation; for the nervous anticipation and the disadvantages scale, a higher score meant more negative effects. The score from the visual analogue scale was the number of millimetres from the left endpoint to the mark.

To test for an effect of method of elicitation, data obtained by interview or by mailed questionnaire were compared and differences were tested.

The association between subscales and patient characteristics was tested using Mann–Whitney *U*-tests and Kruskal–Wallis analysis of variance. Association between continuous variables was assessed using Spearman's rank-order correlation coefficient. Results are presented by subscale to prevent the problem of multiple comparisons.

### RESULTS

### Response

Of the 90 patients who were approached for an interview, 82 consented (91%). Of the 120 questionnaires mailed, 105 (87.5%) were returned completed. Of the additional sample of 31 questionnaires, mailed to assess an effect of elicitation mode, 25 questionnaires were returned completed (80.6%). This makes a total of 130 questionnaires available for analysis.

In Table 3, patient characteristics for the different groups are given. Twenty-seven patients were interviewed 1 week before the scheduled follow-up visit, 27 patients 2 weeks after a follow-up visit and 28 patients in the middle between two follow-up visits.

### Effect of the follow-up visit on the patient's quality of life

In Table 4 results on the MOS-SF20, the RSCL and the visual analogue scale (VAS) for quality of life are given for the three groups, interviewed at three different times in the course of follow-up. The only differences between the three groups were seen for the physical functioning scale of the MOS-SF20 ($P = 0.02$) and for the visual analogue scale ($P = 0.09$). For both variables, cases interviewed halfway between two visits scored higher than the other two groups. As the physical functioning scale of the MOS pertains to limitations, it is not likely to be affected by a follow-up visit. On the other hand, overall quality of life (VAS) may very well be affected by physical limitations. Indeed, the correlation between physical functioning and the VAS was 0.23 ($P = 0.04$) and if we assessed the association between time of interviewing and VAS, controlling for physical functioning in an analysis of variance, the association disappeared.

© Cancer Research Campaign 1997

**Table 3** Characteristics of the patient groups by elicitation mode

| | Elicitation mode | | |
|---|---|---|---|
| | Interviews ($n$ = 82) | Mailed questionnaires ($n$ = 130) | Total ($n$ = 212) |
| Hospital | | | |
| Leiden University | 48 | 11 | 59 |
| Diaconessen, Leiden | 34 | 14 | 48 |
| Leyenburgh, The Hague | | 55 | 55 |
| Medical Center Alkmaar | | 50 | 50 |
| Mean age (s.d.) | 68 (12) | 68 (11) | 68 (11) |
| Number of men (%) | 34 (42) | 73 (56%) | 107 (51%) |
| Frequency of follow-up | | | |
| 3-Monthly | 35 (43%) | 29 (25%) | 64 (32%) |
| 6-Monthly | 42 (51%) | 65 (55%) | 107 (54%) |
| Yearly | 5 (6%) | 24 (20%) | 29 (15%) |
| Living situation | | | |
| Living alone | 21 (26%) | 33 (26%) | 54 (26%) |

**Figure 1** Median scores on the attitude scales, by elicitation mode: interview ($n$ = 66) vs mailed questionnaire ($n$ = 103). The differences were statistically significant for the factor anticipation ($P < 0.001$).

### Attitudes towards and strength of preference for follow-up

### *Attitudes towards follow-up*

In Table 2 the scores on the items of the follow-up questionnaire are given. To the large majority of patients, follow-up conveys a sense of security and consequently they do not prefer to have follow-up visits less frequently. Half of the patients (46%) state that the advantages outweigh the disadvantages to a large extent. It is remarkable that over half of the patients do not indicate that they are nervous or that they dread the follow-up visits. Very few patients feel that they cannot ask things or discuss problems with their physician, and very few would prefer follow-up by their family physician.

In Figure 1, the scores on the follow-up scales are given for the interviews and the mailed questionnaires. We found significant differences between the scores from interviews and those from mailed questionnaires. In general, scores from the interviews were more positive, suggesting that the interviewed patients gave more socially desirable answers. Scores from the additional questionnaires, sent to patients from the two hospitals at which the interviews had been conducted, were closer to the scores from the questionnaires in the other two hospitals than to those from the interviews in the same hospitals. Thus this discrepancy between scores from interviews and those from questionnaires seems to be an effect of mode, not of region or hospital.

In general, patients have a positive attitude regarding follow-up. Scores on the two positive scales (communication with the physician and reassurance) are much higher than those on the negative ones (nervous anticipation and general disadvantages). Patients regard communication with the physician as positive and obtain a sense of reassurance from follow-up, whereas they do not perceive the disadvantages to be large, nor do they indicate feeling much nervous anticipation.

A positive correlation was seen between communication with the physician and reassurance ($r_s = 0.32$, $P < 0.001$) as well as between nervous anticipation and disadvantages ($r_s = 0.36$, $P < 0.001$).

**Table 4** Quality of life in patients interviewed at three different times in relation to the follow-up visit: 1 week before the visit, 2 weeks after the visit and halfway between two visits [medians (interquartile range) and P-value for test of difference (Kruskal–Wallis one-way ANOVA)]

| | One week before ($n$ = 27) | Two weeks after ($n$ = 27) | Halfway ($n$ = 28) | $P$-value |
|---|---|---|---|---|
| RSCL | | | | |
| Physical | 0.18 (0.23) | 0.27 (0.50) | 0.16 (0.28) | 0.32 |
| Psychological | 0.13 (0.38) | 0.25 (0.75) | 0.13 (0.50) | 0.43 |
| MOS-SF20 | | | | |
| Health perceptions | 80 (15) | 80 (15) | 80 (15) | 0.49 |
| Physical | 83.3 (33.3) | 83.3 (33.3) | 100 (14.6) | 0.02 |
| Mental | 92 (20) | 84 (32) | 90 (20) | 0.70 |
| Social | 100 (0) | 100 (0) | 100 (0) | 0.30 |
| Role | 100 (0) | 100 (0) | 100 (0) | 0.11 |
| Pain | 0 (25) | 0 (50) | 0 (18.8) | 0.39 |
| Visual analogue scale | 86 (40) | 84 (22) | 92.5 (17) | 0.09 |
| Fear of recurrence | 16.7 (33.3) | 22.2 (22.2) | 22.2 (33.3) | 0.48 |

© Cancer Research Campaign 1997

### Association with patient characteristics, medical history and quality of life

The only statistically significant association with sociodemographic characteristics was that patients living alone had a more positive attitude towards follow-up than those who were married or were living with a sibling or friend (Figure 2). They indicated a better communication with the physician ($P < 0.001$), experienced a stronger sense of reassurance because of follow-up ($P = 0.02$) and experienced less general disadvantages of follow-up ($P = 0.004$) than patients not living alone. As more women than men lived alone, and women felt a somewhat higher sense of reassurance (median scores 75 and 67, $P = 0.09$), we analysed this relationship more closely. An interaction emerged between gender and living situation: the higher reassurance score for those living alone only existed for women [medians for those living alone and for those not living alone were 79 and 71 respectively ($P = 0.02$); for men, the corresponding ranks were 71 and 68 ($P = 0.55$)].

Patients who had yearly follow-up visits indicated more general disadvantages of follow-up than patients who had 3- or 6-monthly appointments (medians 22 vs 11 and 11 respectively, $P = 0.02$). This was predominantly as a result of these patients indicating more often that the examinations at follow-up are burdensome and that follow-up reminds them of their disease whereas they would rather not think about it. Fifty-nine per cent of these patients indicated examinations to be burdensome to some extent, compared with 40% and 42% in the 3-monthly and 6-monthly group respectively. Thirty-five per cent indicated that the visit reminded them too much of their disease, compared with 23% in the other two groups.

The reassurance scale was associated with interview time in relation to follow-up. Reassurance was lower in the group interviewed midway between the two visits than in the other two groups (medians 67 vs 83 and 83 respectively, $P = 0.08$).

The only attitude scale that correlated with quality of life variables was the nervous anticipation scale. It correlated with the psychological distress scale of the RSCL ($r_s = 0.33$, $P < 0.001$) and with fear of recurrence ($r_s = 0.27$, $P < 0.001$). Patients who experienced more psychological distress and more fear of a recurrence felt more nervous before follow-up visits.

### Strength of preference for follow-up

Patients were reluctant to switch to the 'no follow-up' strategy. The great majority of patients (63%) would not switch at all and preferred follow-up irrespective of the chances of early detection (see Table 5). Of the other patients, 10% would only switch to 'no follow-up' if the chances of early detection were more favourable than in the 'follow-up' strategy. Only one-fifth of patients switched to 'no follow-up' in the case of equal chances of early detection.

**Figure 2** Association between living situation and attitude scales: median scores for patients living alone ($n$ = 42) and those not living alone ($n$ = 126)

**Table 5** Treatment preference question: number (%) of patients who switched from 'follow-up' to 'no follow-up' at various chances of early detection of a recurrence in the 'no follow-up' strategy (starting point in the 'no follow-up' strategy was a 40% chance of early detection; the hypothetical chance of early detection for 'follow-up' was held constant at 80%)

| | *n* | (%) |
|---|---|---|
| Switched at early detection rate of: | | |
| ≤ 80% | 4 | (5) |
| 80% | 18 | (22) |
| > 80% | 7 | (9) |
| Never switched to 'no follow-up' | 52 | (64) |
| Total | 81 | (100) |

For those who were more strongly in favour of follow-up, i.e. those who always preferred follow-up or who switched only at more favourable chances in the 'no follow-up' strategy ($n$ = 59), reassurance was more important than for those who switched at equal or somewhat lower chances in the 'no follow-up' strategy ($n$ = 22); median-score values were 83 and 67 respectively ($P = 0.02$). In addition, they reported less nervous anticipation (median values 3 and 13, $P = 0.09$). No association was seen with living situation, fear of recurrence or actual frequency of follow-up visits.

### DISCUSSION

We have not found a clear effect of the follow-up visit on the quality of life of patients with colorectal cancer. The only differences between the three different interview times were seen for physical functioning and overall quality of life. For both variables, the group interviewed midway between two follow-up visits scored higher than the other two groups. However, we did not expect physical limitations to be caused by a follow-up visit, and we did expect quality of life to be affected by physical limitations. Indeed, the two variables correlated positively. When the association between time of interview and overall quality of life was adjusted for physical functioning, the association disappeared. We did, however, expect to find differences in psychological functioning or distress, but this was not the case. Our findings are in contrast with an earlier study at our hospital, which found that the group interviewed midway between two visits showed less distress on the RSCL (Kiebert et al, 1993). This may have been due to the design of our study. For logistic reasons it was not possible to assess the effect in a longitudinal way, which would have been the most powerful solution. However, such an assessment shows only an effect of the visit itself and not the effect of being in a follow-up programme. A randomized study would be the only way to compare patients within and outside a follow-up programme. The GIVIO study (1994) is one such study in breast cancer. In this study, no differences were seen between intensive and minimalist follow-up, but both groups were under some form of surveillance. Moreover, the prospects are different for breast and colorectal cancer once the disease has metastasized. However, one thing that can be concluded from both studies at our institute is that, within

© Cancer Research Campaign 1997

the context of follow-up, a visit does not seem to have a detrimental effect on quality of life. The only effect, if any, seems to be a temporary increase in quality of life following a visit (particularly a visit during which no signs of recurrence were seen).

We have found that patients with colorectal cancer who are under regular surveillance have a very positive attitude towards follow-up. They indicate a positive communication with the physician, a strong sense of reassurance, little nervous anticipation and few other disadvantages. As very few patients had dropped out of the follow-up programme, either out of their own will or because of organizational problems, our data are likely to be representative of the colorectal cancer population submitted to follow-up in The Netherlands. It should be kept in mind, however, that some of our results may be less applicable to other countries, with for instance more travel time to the clinic. We found an indication that interviewed patients give more socially desirable answers (Cook et al, 1993): the results from the interviews were even slightly more positive than those from the mailed questionnaires.

Both a previous study at our institute (Kiebert et al, 1993) and the GIVIO study (1994) report a positive attitude of cancer patients towards follow-up, and patients expressed a strong preference for routine visits. To some extent these findings are not unexpected. It may be difficult to elicit a negative opinion on follow-up from patients who feel that non-compliance may jeopardize their life expectancy. Most of the physicians will have presented follow-up as necessary and will have expressed no doubts to the patients as to the value of the follow-up. Therefore, such positive attitudes should be interpreted with caution. It may be a psychological mechanism through which patients manage to keep their motivation to adhere to the follow-up protocol. This could explain why subjects interviewed midway between two visits indicate fewer positive effects and more disadvantages. They do not have to justify the visit they are about to have, or have just had. It should also be noted that patients who had yearly follow-up visits indicated more disadvantages of follow-up. As time goes by and prognosis improves (resulting in a less frequent follow-up schedule), the negative aspects become more salient. One of these aspects is the examination at follow-up, which will include relatively more often burdensome coloscopy. On the other hand, follow-up reminds patients of their cancer when they would perhaps otherwise think less and less often about their disease.

It is debatable whether the positive attitude of a patient, added to the temporary increase in well-being after a follow-up visit (Kiebert et al, 1993), is sufficient grounds for routine follow-up. A large proportion of health care resources is being targeted at an intervention that may not have the highest priority in an era of scarcity. Moreover, reassurance may also be seen as a negative factor in cases for which follow-up is not effective; this could lead to a false sense of security and to diagnostic delay (Isbister, 1988). To assess whether follow-up is overall a useful procedure, its cost-effectiveness should be assessed, taking both survival and quality of life into account. In such an analysis, the benefits of follow-up in terms of life expectancy can be weighed against the disadvantages in terms of earlier diagnosis of incurable recurrences in some patients (the diagnosis of incurability being advanced by the so-called lead time).

Should one ever consider abandoning follow-up, based, for instance, on considerations of cost-effectiveness, our results shed light on the psychological barriers that may be encountered. As our preference question showed, it is very hard for patients to even consider the idea that 'no follow-up' may be an alternative. The common opinion in the population seems to be that the diagnosis of cancer implies routine follow-up, and thus anxiety may be caused if follow-up were simply abandoned. However, as Brada (1995) remarked, perhaps patients may not be too distressed about omitting routine follow-up visits, if armed with correct information of the lack of value of clinical follow-up. Nevertheless, in these circumstances, a plea should be made for a careful process of abolishing the visits. A subgroup that may need special attention in this respect seems to be the group of patients living alone. We found a striking association between living situation and attitude towards follow-up, with patients living alone indicating greater advantage overall.

An alternative to routine follow-up by the surgeon or oncologist may be follow-up by either nurse clinics or by family physicians (James et al, 1994; Williams, 1994; Wyatt and Aitken, 1994; Brada, 1995). In particular, if the diagnostic work-up consists only of blood tests, such as CEA, both might be reasonable alternatives. Additionally, identifying patients with low risk of anticipated medical complications and providing a structure for a phone-based outpatient consultation may be one way of retaining patient contact with oncology services and reducing hospital activity (James et al, 1994). Very few of our patients indicated a preference for follow-up by the family physician, partly because of the positive evaluation of the communication with their specialist. (Patients who preferred follow-up by their family physician showed slightly, though not significantly, lower scores on the communication scale; data not shown.) Therefore, in these circumstances, it should be made clear to patients that their family physician does have the expertise needed and that follow-up by the surgeon or oncologist does not guarantee a better outcome.

### ACKNOWLEDGEMENTS

The authors thank Professor Dr K Welvaart and Dr HJ Keizer (Leiden University Hospital), Dr H van Slooten (Diaconessen Hospital Leiden) and Dr P de Ruiter (Medical Center Alkmaar) for the permission to interview their patients, J Molenaar (Leiden University Hospital) and P de Mooij (Medical Center Alkmaar) for their valuable help in the selection of the patients, M Heyboer for her assistance in interviewing and for data processing, Dr GM de Bock for her helpful comments on an earlier version of this paper and, last but not least, the patients for their cooperation. This study was supported by the Netherlands Insurance Board (Grant OG 91/051).

### APPENDIX: DEVELOPMENT OF THE ATTITUDES TOWARDS FOLLOW-UP QUESTIONNAIRE

In the previous study (Kiebert et al, 1993), a questionnaire had been used consisting of ten items that pertained to patients' attitudes regarding regular follow-up. In a factor analysis based on the data from that study, three (orthogonal) factors or subscales had been found: a factor pertaining to the communication with the surgeon, a factor tapping feelings of nervous anticipation caused by the follow-up visit and a factor relating to some general disadvantages of follow-up. To improve the reliability (internal consistency) of the questionnaire, we added six items, one pertaining to nervous anticipation, three to general disadvantages and two to communication. A more stable 4-factor solution was found: the first two original scales were recovered (communication and nervous anticipation, explaining 21% and 9% of the variance respectively), a third factor referred to feelings of reassurance (explaining 14% of variance) and a final pertained to the disadvantages of follow-up (5% of variance).

© Cancer Research Campaign 1997

### REFERENCES

Boyd NF, Sutherland HJ, Heasman KZ, Tritchler DL and Cummings BJ (1990) Whose utilities for decision analysis? *Med Decis Making* **10**: 58–67

Brada M (1995) Is there a need to follow-up cancer patients? *Eur J Cancer* **31A**: 655–657

Broyn T and Froyen J (1982) Evaluation of routine follow up after surgery for breast carcinoma. *Acta Chir Scand* **148**: 401–404

Bruinvels DJ (1995) *Follow-up of patients with colorectal cancer*. (PhD-thesis). University of Leiden: Leiden, The Netherlands

Bruinvels DJ, Stiggelbout AM, Kievit J, Van Houwelingen JC, Habbema JDF and Van De Velde CJH (1994) Follow-up of patients with colorectal cancer: a meta-analysis. *Ann Surg* **219**: 174–182

Deveney KE and Way LW (1984) Follow-up of patients with colorectal carcinoma. *Am J Surg* **148**: 717–722

Cook DJ, Guyatt GH, Juniper E, Griffith L, Mcilroy W, Willan A, Jaeschke R and Epstein R (1993) Interviewer versus self-administered questionnaires in developing a disease-specific, health-related quality of life instrument for asthma. *J Clin Epidemiol* **46**: 529–534

GIVIO Investigators (1994) Impact of follow-up testing on survival and health-related quality of life in breast cancer patients: a multicenter randomized controlled trial. *JAMA* **271**: 1587–1592

Haes JCJM de, Knippenberg FCE Van and Neijt JP (1990) Measuring psychological and physical distress in cancer patients: structure and application of the Rotterdam Symptom Checklist. *Br J Cancer* **62**: 1034–1038

Isbister WH (1988) The follow-up of patients following surgery for colorectal cancer – a personal view. *Ann Acad Med* **17**: 66–71

James ND, Guerrero D and Brada M (1994) Who should follow up cancer patients? Nurse specialist based outpatient care and the introduction of a phone clinic system. *Clin Oncol* **6**: 283–287

Kiebert GM, Welvaart K and Kievit J (1993) Psychological effects of routine follow-up on cancer patients after surgery. *Eur J Surg* **159**: 601–607

Llewellyn-Thomas HA, Thiel EC and Clark RM (1989) Patients versus surrogates: whose opinion counts on ethics review panels? *Clin Res* **37**: 501–505

Loprinzi CL (1995) Follow-up testing for curatively treated cancer survivors. *JAMA* **272**: 1877–1878

Rutgers EJT (1986) *De nacontrole van patienten behandeld voor borstkanker. The follow-up of patients treated for breast cancer*. (PhD thesis). University of Amsterdam: Amsterdam, The Netherlands.

Stewart AL, Hays RD and Ware JE (1988) The MOS short-form general health survey. *Med Care* **26**: 724–735

Stiggelbout AM, Kiebert GM, Kievit J, Leer JWH, Habbema JDF and Haes JCJM de (1995) The 'utility' of the Time Trade-Off method in cancer patients: feasibility and proportional trade-off. *J Clin Epidemiol* **48**: 1207–1214

Sugarbaker PH, Gianola FJ, Dwyer A and Neuman NR (1987) A simplified plan for follow-up of patients with colon and rectal cancer supported by prospective studies of laboratory and radiologic test results. *Surgery* **102**: 79–87

Virgo KS, Vernava AM, Longo WE, McKirgan LW and Johnson FE (1995) Cost of patient follow-up after potentially curative colorectal cancer treatment. *JAMA* **273**: 1837–1841

Williams PT (1994) The role of family physicians in the management of cancer patients. *J Cancer Educ* **9**: 67–72

Wyatt JP and Aitken RJ (1994) Evaluation of hospital and general practice follow-up after surgery for colorectal cancer. *Br J Surg* **81**: 145

 *© Cancer Research Campaign 1997*


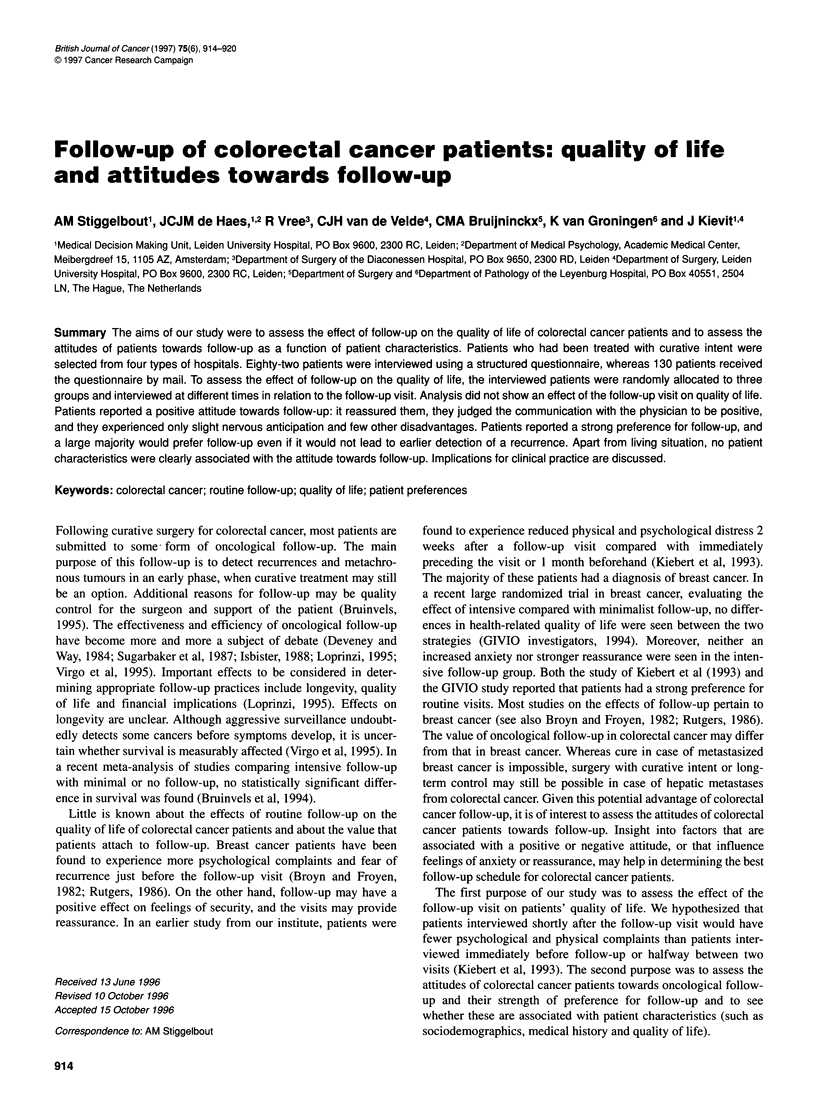

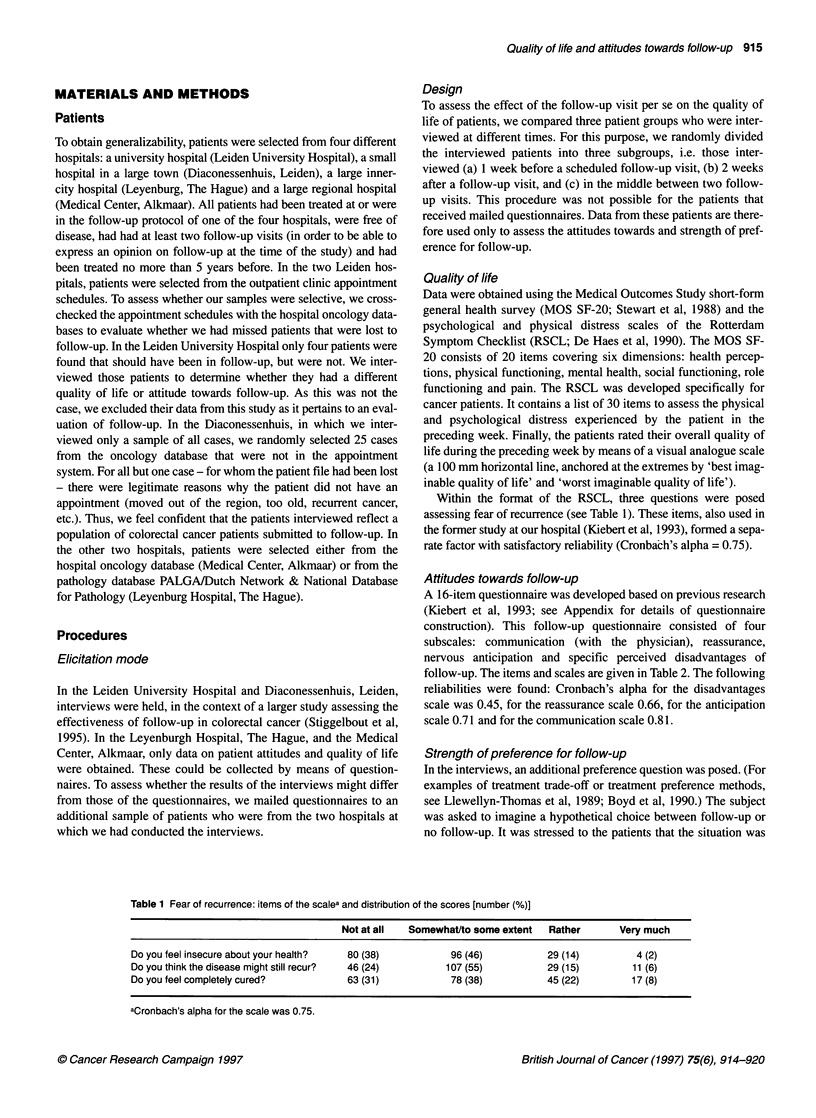

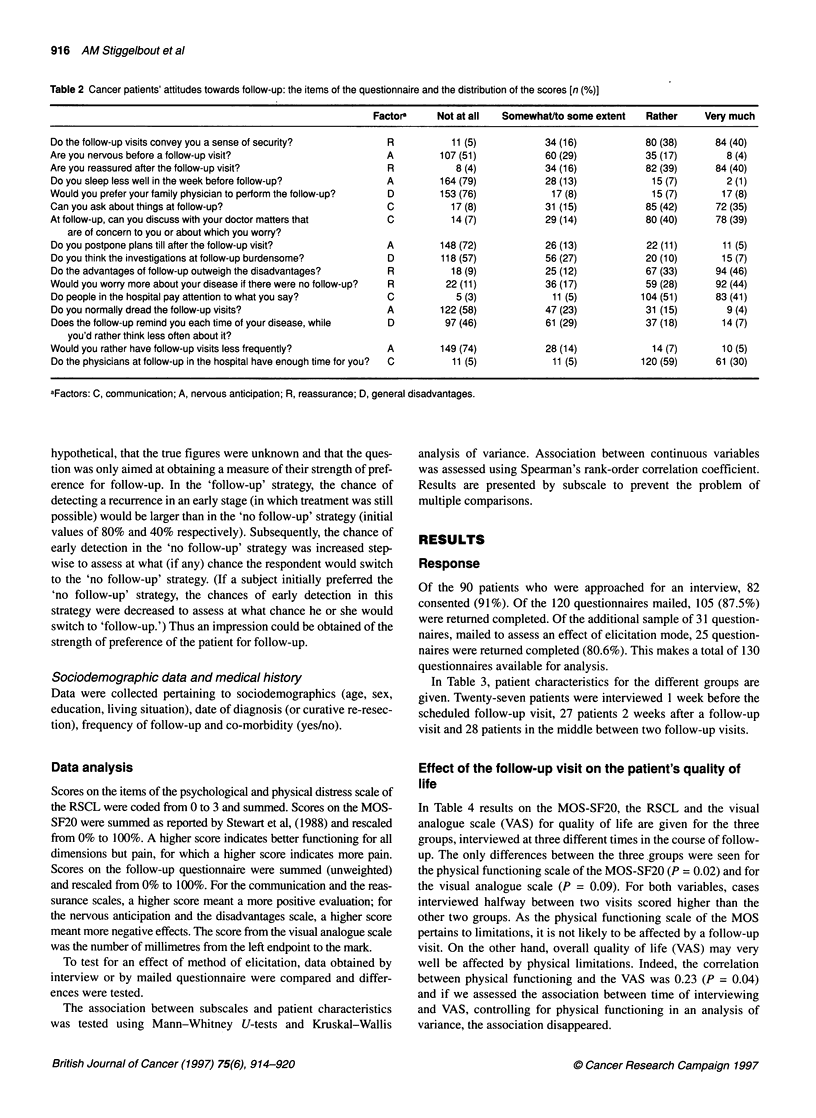

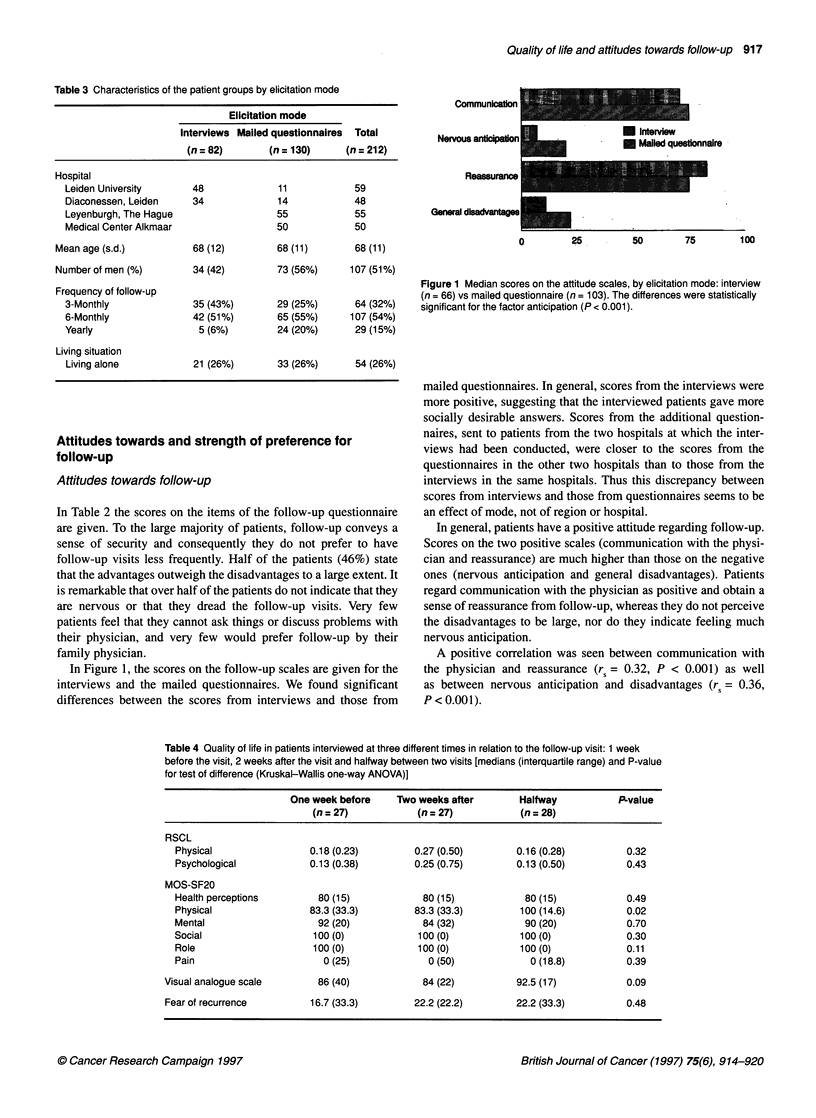

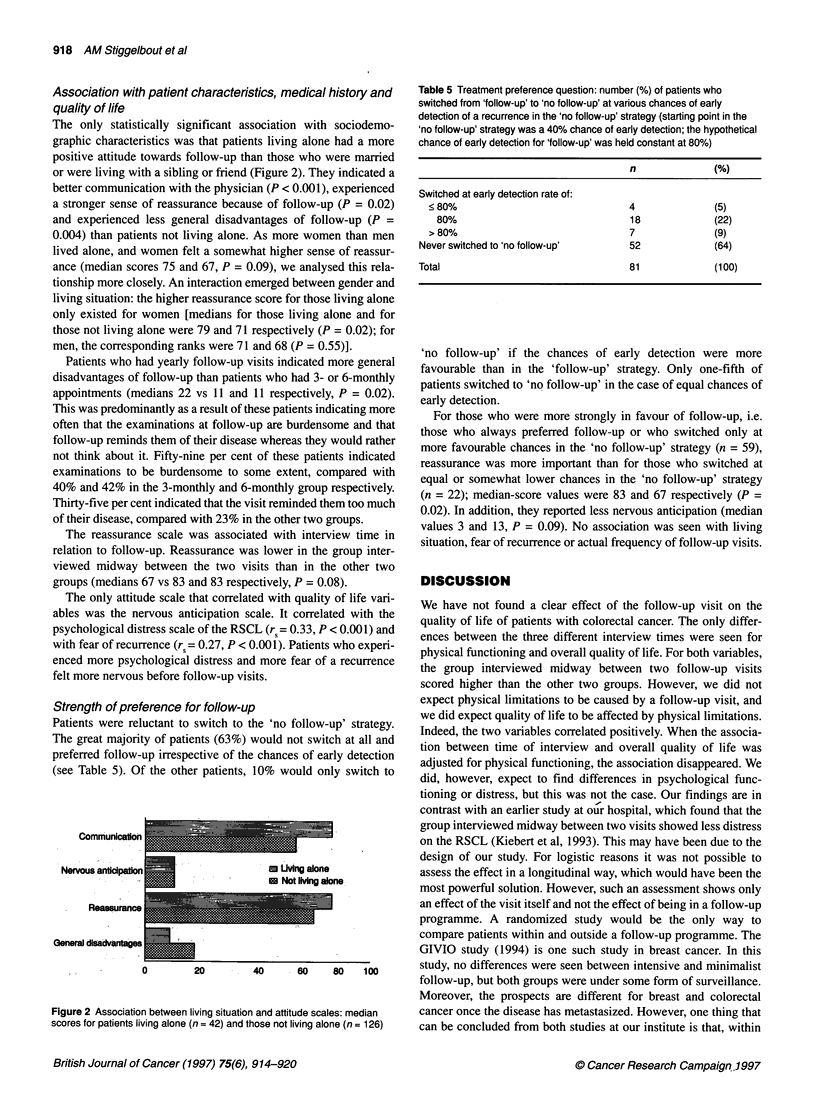

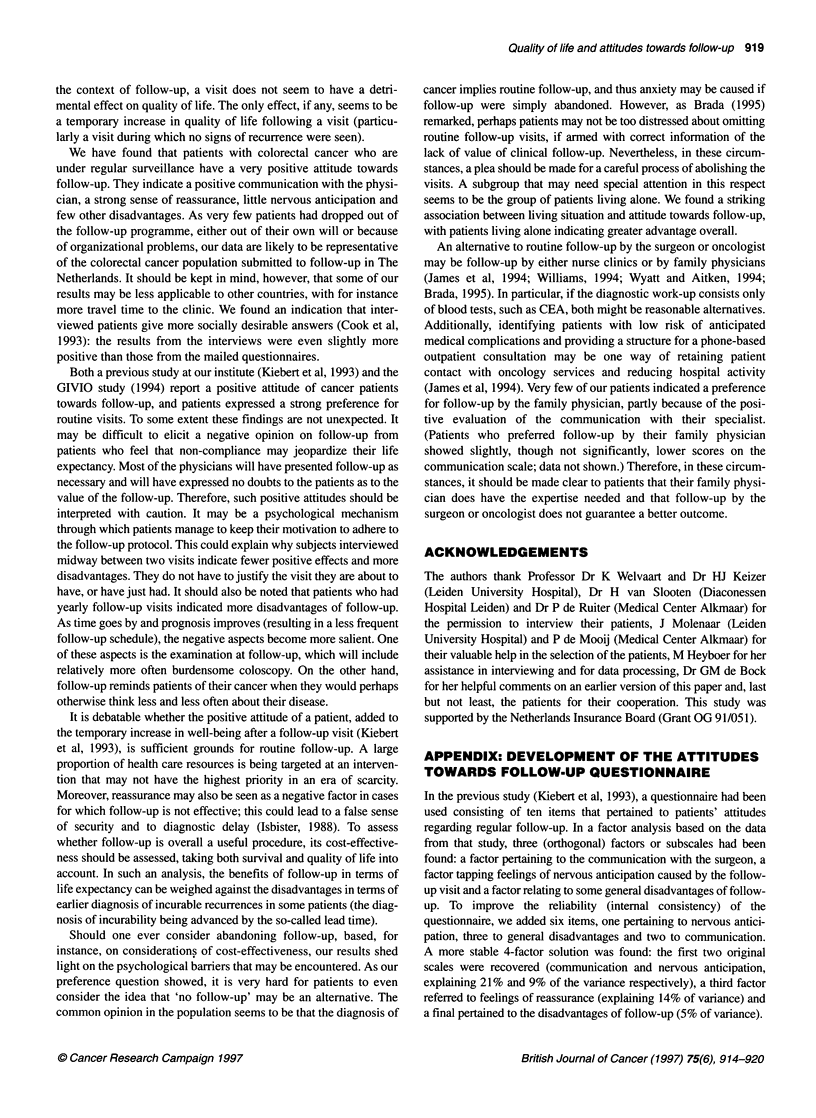

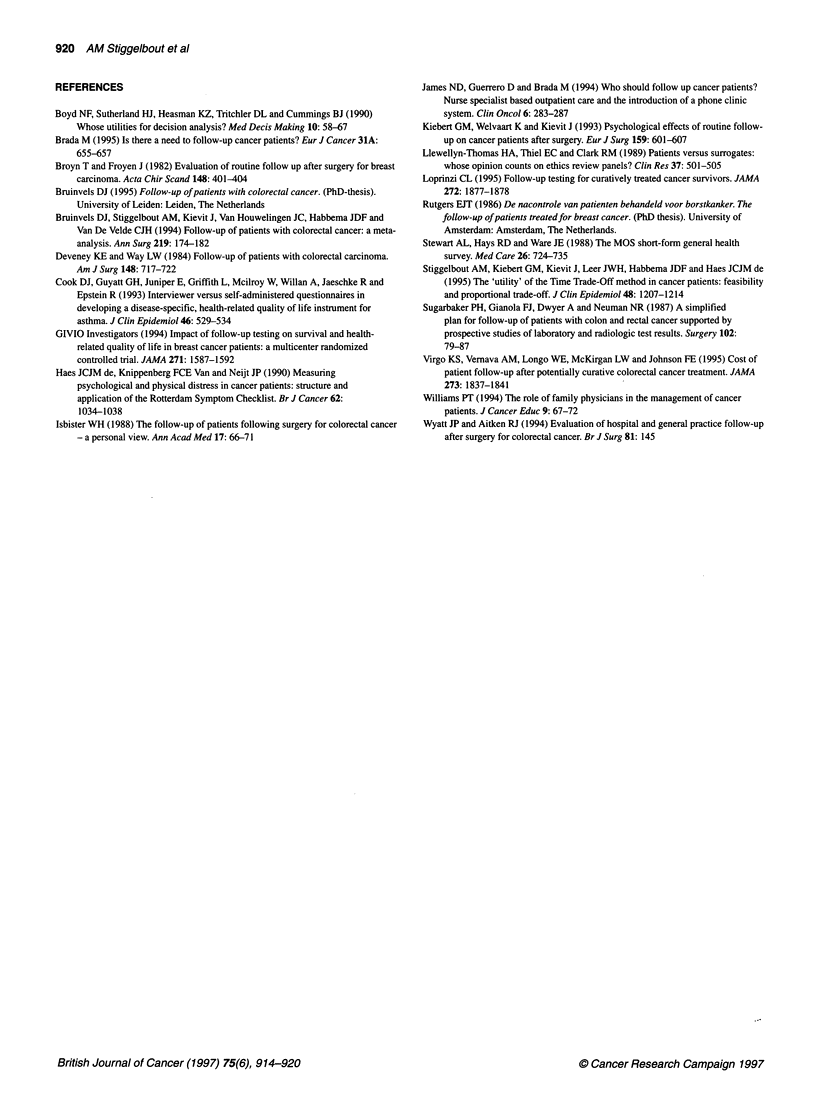


## References

[OCR_00762] Boyd N. F., Sutherland H. J., Heasman K. Z., Tritchler D. L., Cummings B. J. (1990). Whose utilities for decision analysis?. Med Decis Making.

[OCR_00766] Brada M. (1995). Is there a need to follow-up cancer patients?. Eur J Cancer.

[OCR_00778] Bruinvels D. J., Stiggelbout A. M., Kievit J., van Houwelingen H. C., Habbema J. D., van de Velde C. J. (1994). Follow-up of patients with colorectal cancer. A meta-analysis.. Ann Surg.

[OCR_00770] Brøyn T., Frøyen J. (1982). Evaluation of routine follow-up after surgery for breast carcinoma.. Acta Chir Scand.

[OCR_00787] Cook D. J., Guyatt G. H., Juniper E., Griffith L., McIlroy W., Willan A., Jaeschke R., Epstein R. (1993). Interviewer versus self-administered questionnaires in developing a disease-specific, health-related quality of life instrument for asthma.. J Clin Epidemiol.

[OCR_00783] Deveney K. E., Way L. W. (1984). Follow-up of patients with colorectal cancer.. Am J Surg.

[OCR_00805] Isbister W. H. (1988). The follow-up of patients following surgery for colorectal cancer--a personal view.. Ann Acad Med Singapore.

[OCR_00809] James N. D., Guerrero D., Brada M. (1994). Who should follow up cancer patients? Nurse specialist based outpatient care and the introduction of a phone clinic system.. Clin Oncol (R Coll Radiol).

[OCR_00814] Kiebert G. M., Welvaart K., Kievit J. (1993). Psychological effects of routine follow up on cancer patients after surgery.. Eur J Surg.

[OCR_00818] Llewellyn-Thomas H. A., Thiel E. C., Clark R. M. (1989). Patients versus surrogates: whose opinion counts on ethics review panels?. Clin Res.

[OCR_00822] Loprinzi C. L. (1995). Follow-up testing for curatively treated cancer survivors. What to do?. JAMA.

[OCR_00831] Stewart A. L., Hays R. D., Ware J. E. (1988). The MOS short-form general health survey. Reliability and validity in a patient population.. Med Care.

[OCR_00835] Stiggelbout A. M., Kiebert G. M., Kievit J., Leer J. W., Habbema J. D., De Haes J. C. (1995). The "utility" of the Time Trade-Off method in cancer patients: feasibility and proportional Trade-Off.. J Clin Epidemiol.

[OCR_00840] Sugarbaker P. H., Gianola F. J., Dwyer A., Neuman N. R. (1987). A simplified plan for follow-up of patients with colon and rectal cancer supported by prospective studies of laboratory and radiologic test results.. Surgery.

[OCR_00847] Virgo K. S., Vernava A. M., Longo W. E., McKirgan L. W., Johnson F. E. (1995). Cost of patient follow-up after potentially curative colorectal cancer treatment.. JAMA.

[OCR_00852] Williams P. T. (1994). The role of family physicians in the management of cancer patients.. J Cancer Educ.

[OCR_00856] Wyatt J. P., Aitken R. J. (1994). Evaluation of hospital and general practice follow-up after surgery for colorectal cancer.. Br J Surg.

[OCR_00799] de Haes J. C., van Knippenberg F. C., Neijt J. P. (1990). Measuring psychological and physical distress in cancer patients: structure and application of the Rotterdam Symptom Checklist.. Br J Cancer.

